# Contributions of the Biofilm Matrix to *Candida* Pathogenesis

**DOI:** 10.3390/jof6010021

**Published:** 2020-02-03

**Authors:** Jeniel E. Nett, David R. Andes

**Affiliations:** Departments of Medicine and Medical Microbiology & Immunology, University of Wisconsin-Madison, Madison, WI 53706, USA; dra@medicine.wisc.edu

**Keywords:** *Candida*, matrix, biofilm, immunity, drug resistance

## Abstract

In healthcare settings, *Candida* spp. cause invasive disease with high mortality. The overwhelming majority of cases are associated with the use of critically-needed medical devices, such as vascular catheters. On the surface of these indwelling materials, *Candida* forms resilient, adherent biofilm communities. A hallmark characteristic of this process is the production of an extracellular matrix, which promotes fungal adhesion and provides protection from external threats. In this review, we highlight the medical relevance of device-associated *Candida* biofilms and draw attention to the process of *Candida*-biofilm-matrix production. We provide an update on the current understanding of how biofilm extracellular matrix contributes to pathogenicity, particularly through its roles in the promoting antifungal drug tolerance and immune evasion.

## 1. What Is the Health Impact of *Candida* Biofilms?

*Candida* spp. are the leading cause of invasive fungal infection in the clinical setting [1]. A recent multistate point prevalence survey of hospitalized patients found *Candida* spp. to be the group of microorganisms accounting for the greatest number of bloodstream infections (22%) [1]. As invasive candidiasis carries the highest mortality rate among all nosocomial pathogens (with up to 47% attributable mortality), these infections often lead to devastating consequences [2,3]. Epidemiological studies have linked the presence of medical devices, such as indwelling vascular catheters, to the development of *Candida* bloodstream infection (candidemia) and invasive candidiasis [4]. On artificial devices, *Candida* attaches to the surface and proliferates as adherent microbial communities [5,6,7]. Fungal cells within these biofilms exhibit resistance to all available drug therapies and impair host defenses [8,9,10,11,12,13,14,15,16]. Ultimately, cells from the communities disperse into the bloodstream and disseminate to other organs, including the eyes, heart valves, joints, spleen, kidneys, and liver, too commonly leading to patient’s death [3,17]. Data from the Centers for Disease Control and Prevention show *Candida* spp. to be the most frequent cause of vascular-device-associated bloodstream infection in the hospital setting [1,4,18]. Device removal is recommended for patients with *Candida*-infected medical devices, as mortality rates are even higher if vascular catheters are retained [3,19]. However, removal of vascular access can be difficult for critically-ill patients, and mortality rates remain high even after removal [19].

**Figure 1:**
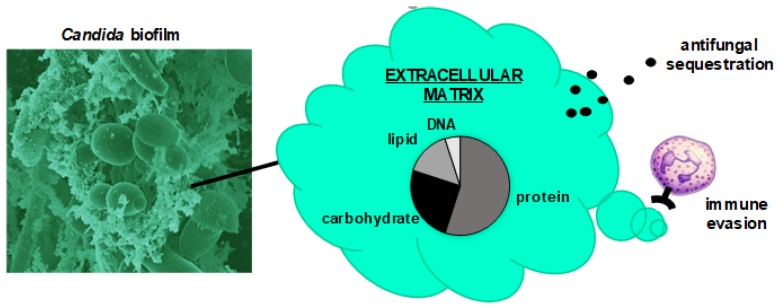
Influence of *Candida* biofilm extracellular matrix on pathogenicity. The scanning electron micrograph shows a *Candida albicans* biofilm consisting of yeast and hyphae encased in an extracellular matrix. This material contains a combination of polysaccharides, proteins, DNA, and lipids. Extracellular matrix contributes to pathogenicity by enhancing drug tolerance and promoting immune evasion.

*Candida albicans*, the most prevalent species of *Candida*, has been employed as the model organism for the study of fungal biofilms [20,21,22,23,24,25,26,27,28,29,30,31,32]. As biofilms form, *C. albicans* frequently undergoes filamentation, producing elongated pseudohyphae and hyphae, in addition to maintaining cells in the yeast morphology [33]. The extent of this morphologic transition appears to vary among *C. albicans* strains and clinical niches. In addition to *C. albicans,* the importance of non-*albicans* species, including *C. tropicalis*, *C. parapsilosis*, and *C. glabrata* has become increasingly recognized [7,21,33,34,35,36,37,38]. Similarly, the recently emergent species *C. auris* readily forms biofilms on artificial materials, which likely accounts for the predilection of this species to cause disease in patients with indwelling medical devices [39,40,41,42,43,44,45]. Many of the non-*albicans Candida* spp. lack the capacity for filamentation and instead form biofilms comprised entirely of yeast cells [46]. However, biofilms formed by all *Candida* spp. involve the production of a matrix, which is an extracellular adherent polymeric material that defines biofilms [20,47,48,49]. In addition to its roles in adhesion and cohesion, this material also accounts for the vast majority of the tolerance of antifungals and resistance to host defenses that has been observed for *Candida* biofilms (Figure 1) [11,13,20,50].

## 2. What Is the Composition of *Candida* Biofilm Matrix?

The most detailed characterizations of *Candida* biofilm matrix have examined *C. albicans* biofilms during in vitro growth [29,51,52]. These investigations show that the matrix of mature biofilms contains a variety of macromolecules, including protein (55%), carbohydrate (25%), lipid (15%), and DNA (5%). Although the most abundant matrix component is protein, very little is known about the function of the individual proteins [29,32,53]. The finding of various metabolism-related proteins suggests that the matrix may function to degrade extracellular biopolymers as an energy source or as a mechanism of dispersion [32,53]. Although these hypotheses have not been tested specifically for *Candida*, the role of biofilm matrix as an extracellular digestive system has been described for bacterial biofilms [54,55,56,57,58].

The polysaccharide component of *Candida* biofilms contains building blocks similar to those found in the cell wall [29,43,59]. However, the macromolecular structures of the polysaccharides vary considerably between the cell wall and the extracellular matrix. For example, in the extracellular matrix of *C. albicans*, one abundant high-molecular-weight component consists of approximately 12,000 residues of α-1,2-branched α-1,6 mannan. These polysaccharide residues are nearly 10-fold greater than the mannans found in the *C. albicans* cell wall. Furthermore, they assemble with linear β-1,6 glucan in the extracellular space, forming a mannan–glucan complex [27,29]. Other *Candida* spp., including *C. tropicalis*, *C. parapsilosis*, *C. glabrata*, and *C. auris*, also produce a polysaccharide-rich matrix during biofilm growth [43,59]. Similar to *C. albicans*, these biofilms also contain mannan complexed with glucan. These polysaccharides sequester drugs and contribute to the resistance of biofilms to antifungals, as described in the following section [27,43].

Less abundant macromolecules of the biofilm matrix include lipids and nucleic acid [29,60,61]. The extracellular matrix of *C. albicans* biofilms contains a variety of lipids, including phospholipids (predominately phosphatidylcholine and phosphatidylethanolamine), sphingolipids, and eicosanoids [29,60,61]. The role of these lipids during *Candida* biofilm formation remains largely unexplored. The nucleic acid component of *C. albicans* biofilms appears to be comprised of non-coding DNA, which is anticipated to provide a structural scaffold and participate in protection from external insults, including some antifungal drugs [50,60,62].

Maybe not surprisingly, the extracellular matrix of *C. albicans* biofilm varies considerably between in vitro and in vivo conditions [28,29]. For example, during in vivo conditions, host proteins contribute to the extracellular biofilm matrix, accounting for >95% of the matrix proteome [28]. The composition of incorporated proteins varies by environmental niche (e.g., saliva, blood, urine). Little is known about the role of many of these host proteins. However, a subset of proteins appears to represent the host’s immune response to the biofilm [28]. Given the abundance of host proteins in biofilm matrix, further understanding of their role in biofilm pathogenicity will be of interest.

## 3. How Is *Candida* Biofilm Matrix Produced?

*C. albicans* has served as the model organism for discovery of pathways and regulators of biofilm matrix biogenesis [27,31,63,64,65,66]. During biofilm formation, *C. albicans* produces extracellular vesicles that are distinct from those manufactured during planktonic growth [66]. These 30-200 nm structures consist of lipid bilayers encasing protein, nucleic acid, lipid, and carbohydrate cargo. As *C. albicans* biofilms mature, cells release vesicles into the extracellular space and the accompanying cargo incorporates in the matrix. The importance of vesicle delivery in matrix biogenesis is highlighted by analysis of *C. albicans* mutants with disruption of key components of this pathway. Mutants defective in orthologs of endosomal sorting complexes required for transport (ESCRT) subunits fail to produce characteristic extracellular vesicles and do not manufacture a mature extracellular matrix during biofilm growth [66].

In addition to depositing in the extracellular matrix and providing a structural function, vesicle cargo proteins also remain enzymatically active. During biofilm formation, enzymes classically involved in cell wall biosynthesis are transported to the extracellular space and contribute to the assembly of mannans and glucans in the matrix [27]. Disruption of genes involved in either mannan pathways (*ALG11, MNN9, MNN11, VAN1, MNN4-4, PMR1*, and *VRG4*) or β-1,6 glucan pathways (*BIG1* and *KRE5*) similarly impairs the deposition of both types of polysaccharides into the matrix. However, the ability of these mutants to complement each other during biofilm co-culture shows that these enzymes are active in the extracellular matrix and are critical for matrix assembly. Biofilms formed by other *Candida* spp., including *C. tropicalis*, *C. parapsilosis*, *C. glabrata*, and *C. auris*, also involve extracellular synthesis and modification of polysaccharides [43,59]. However, the genes involved in extracellular modification of mannan and glucan varies among species, likely accounting for their observed differences in polysaccharide side chains [59].

Several investigations have identified and characterized regulators of matrix production during *C. albicans* biofilm growth. These pathways appear to interface with stress response pathways during planktonic growth. For example, the molecular chaperone Hsp90 is required for mature matrix production [9]. However, in contrast to its role in stabilizing calcineurin under planktonic stress conditions, Hsp90 modulates matrix production through a separate, calcineurin-independent pathway during biofilm growth. Studies have also revealed two transcription factors involved in regulating matrix production for *C. albicans* biofilms, *ZAP1* and *RLM1* [31,63]. Zap1 serves as a negative regulator of matrix production, as genetic deletion leads to increased extracellular matrix production, presumably due to the upregulation of glucoamylases [63]. Rlm1 acts as positive regulator of extracellular matrix production, likely through the transcription of *SMI1*, which modulates extracellular glucan production during biofilm growth. Other transcriptional regulators of *C. albicans* biofilm development are also likely to influence extracellular matrix production [67].

## 4. How Does the Extracellular Matrix of Biofilm Influence Resistance to Antifungals?

One hallmark of *C. albicans* biofilm formation is the capacity of these communities to withstand antifungal concentrations many fold greater than the levels needed to kill *Candida* during planktonic growth [8,9,10,11,12]. As delivery of antifungals at these high concentrations is often unsafe or not feasible, device-associated biofilm infections pose a high risk for treatment failure [3]. Al-Fattani and Douglas first described how the relative abundance of the extracellular matrix correlated with antifungal drug resistance for *Candida* biofilms [22]. By altering the flow conditions, they modulated biofilm matrix production and linked biofilm resistance to abundant matrix production for *C. albicans* and *C. tropicalis.* Additional investigations have since identified correlations between extracellular matrix and tolerance of antifungals for additional *Candida* spp., including *C. parapsilosis*, *C. glabrata*, and *C. auris* [43,59].

Studies to date have shown that antifungal tolerance of *Candida* biofilms correlates most closely with the polysaccharides of the extracellular matrix. The presence of β-1,3 glucan, β-1,6 glucan, and α-1,2-branched α-1,6 mannan contribute to the resistance of *Candida* biofilms to anti-infectives [11,25,27,43,59,68]. The mechanism of the drug––polysaccharide interaction was first described for *C. albicans* biofilm matrix and the azole drug, fluconazole [25]. The glucan and mannan components for the extracellular matrix form a complex that sequesters drugs, likely through non-covalent interactions [27]. Subsequent studies have revealed the involvement of mechanisms of drug sequestration for other *Candida spp.,* including *C. tropicalis, C. parapsilosis*, *C. glabrata*, and *C. auris* [23,43,59]. In addition, the matrix polysaccharides of *Candida* biofilms also sequester other commonly used antifungals, such as amphotericin B, anidulafungin, and flucytosine [11,68]. Furthermore, the abundance of matrix polysaccharides correlates with the ability of *Candida* biofilms to withstand disinfectants and oxidative stressors [69].

For many sites of infection, *Candida* spp. form polymicrobial biofilms with bacteria or other *Candida* spp. [70,71,72]. Examples include biofilms formed in the oropharynx and those found on many indwelling medical devices. In these settings, the extracellular matrix produced by one of the organisms may contribute to collectively protect other organisms within the biofilm. For example, in polymicrobial biofilms formed by *C. albicans* and *Staphylococcus aureus,* the latter exhibits increased resistance to antibiotics, when compared to monomicrobial *Staphylococcus* biofilms [73]. The polysaccharide-rich matrix produced by *Candida* appears to encase both organisms and this material contributes to impaired penetration of the vancomycin, ultimately conferring antibiotic resistance for *Staphylococcus* [74]. Similarly, *Candida* biofilm extracellular matrix also appears to enhance antibiotic resistance for *Escherichia coli* during polymicrobial biofilm growth [75]. However, in other polymicrobial biofilm models, polysacchardides of bacterial origin drive resistance for *Candida* [76]. An example of this scenario is *Streptococcus mutans–C. albicans* biofilms, where bacterially-produced α-glucan enhances the antifungal resistance for *C. albicans* [76].

## 5. Does Biofilm Extracellular Matrix Impact Immune Responses?

Host immune cells respond differently to *Candida* when it is growing as a biofilm or under planktonic conditions [8,13,14,15,16,77,78,79,80]. For example, upon encounter with *C. albicans* biofilm, peripheral blood mononuclear cells exhibit poor antifungal activity and release a cytokine profile distinct from the profile observed in response to planktonic *C. albicans* [16,80]. Similarly, cell culture macrophages appear to have impaired migration in the presence of *C. albicans* biofilms when compared to their motility in response to planktonic *Candida* [79]. Like mononuclear cells, human neutrophils display poor activity against *C. albicans* biofilms, with biofilms exhibiting an up to 5-fold higher resistance to killing when compared to their planktonic counterparts [13,14,15,80]. Similar patterns of resistance to killing by neutrophils have also been observed for biofilms formed by *C. glabrata* and *C. parapsilosis* [77].

As extracellular matrix encases *Candida* biofilm structures, components of this material are among the first to be encountered by leukocytes. Studies examining the role of matrix on leukocyte responses have primarily focused on the interaction of neutrophils with *C. albicans* biofilms [13,14]. Disruption of matrix, through either physical or genetic means, leads to increased killing of *C. albicans* biofilms by neutrophils [13]. The finding that biofilms formed by a mutant strain (*pmr1*∆/∆) deficient in the production of extracellular mannan–glucan are more susceptible to killing by neutrophils suggests a role for these polysaccharide components in immune evasion. The matrix of *C. albicans* biofilms prevents neutrophils from forming neutrophil extracellular traps (NETs), structures of DNA studded with histones and antimicrobial peptides that are important for the killing of many fungi, including *C. albicans* [13,81]. Inhibition of NET formation appears to occur in upstream events, as biofilms also dampen the production of reactive oxygen species, key signaling molecules for NET production [13,14,82]. The *C. albicans pmr1*∆/∆ mutant has also been employed to examine the influence of extracellular matrix on biofilm–macrophage interactions [79]. The finding that genetic disruption of this mannan pathway does not impact macrophage behavior suggests that other matrix components or biofilm properties may be involved in the impaired response of macrophages to biofilm.

## 6. Conclusions

Biofilms formed by *Candida* spp. present a major obstacle for the treatment of invasive candidiasis. During this mode of growth, fungal communities withstand high concentrations of antifungals and resist host responses. The production of extracellular matrix, a defining property of biofilm formation, is critical for providing this protection. Recent studies have defined key matrix polysaccharide components involved in both drug tolerance and immune evasion. Further understanding of these processes may reveal new strategies to combat biofilm infection. While *C. albicans* has served as the model species for many biofilm studies, investigation of other species will be of great interest in light of emerging and drug-resistant *Candida* spp.
